# Supplementary material for: Assembly mechanisms of the bacterial cytoskeletal protein FilP
Source: Life Sci Alliance. 2019 Jun 26;2(3):e201800290. doi: 10.26508/lsa.201800290 (PMC6599971; doi:10.26508/lsa.201800290)
Supplement: Supplementary file 2 [file LSA-2018-00290_TableS1.docx]

| **Table S1** |  |  |  |
| --- | --- | --- | --- |
| **Bacterial strains** | **Describtion** | **Source** | **Name in text and images** |
| *S. coelicolor* |  |  |  |
| WT M145 | Plasmid free prototroph | (Bagchi et al. 2008) | *wt* |
| NA883 | ΔFilP::FRT | (Fuchino et al. 2013) | ∆*filP* |
| LS101 | M145 ΔfilP::FRT attPΦC31pIJ6902(tipAp-His-filP) | This study | ∆*filP tipAp-His-N-filP* |
| LS103 | M145 ΔfilP::FRT attPΦC31pIJ6902(tipAp-filP-His) | This study | ∆*filP tipAp-filP-C-His* |
| *E. coli* |  |  |  |
| DH5α | cloning strain |  |  |
| BL21 (DE3) | expression strain |  |  |
|  |  |  |  |
| **Primers** | **Describtion** | Sequence | **Restriction Enzyme** |
| OLS54 | FilP for expression in E. coli, aa 1 forward | ATTTTCCATGGGCAGCGACACTTCCCCCTACG | NcoI |
| OLS57 | FilP for expression in E. coli, aa 310 reverse | ATATAGAATTCAGCGGGACTGCTGGGCCGGGA | EcoRI |
| OLS61 | FilP for expression in E. coli, aa 71 forward | ATT TTCCATGGGCGCCCGGGTCGAGAAGATC | NcoI |
| OLS109 | C-terminal His-tagged FilP in SCO | ATATACATATGAGCGACACTTCCCCCTA | NdeI |
| OLS118 | C-terminal His-tagged FilP in SCO | ATATAAGATCTTCAGTGGTGATGGTGATGATG GCGGGACTGCTGGGCC | BglII |
| OLS111 | N-terminal His-tagged FilP in SCO | ATATACATATGCATCATCACCATCACCACAGCGACACTTCCCCCTA | NdeI |
| OLS115 | N-terminal His-tagged FilP in SCO | ATATATCTAGATCAGCGGGACTGCTGGGCCGGGA | XbaI |
|  |  |  |  |
| **Plasmids** | **Description** | **Source** |  |
| pETM13-FilP | Non-tagged FilP expression in *E. coli* | This study |  |
| pETM28a-FilP | N-terminally His-tagged FilP expression in *E. coli* | (pNA559, Bagchi et al. 2008) |  |
| pET21b-FilP | C-terminally His-tagged FilP expression in *E. coli* | This study, kindly provided by Nora Ausmees |  |
| pIJ6902 | N-terminally His-tagged FilP expression in *S. coelicolor* | This study |  |
| pIJ6902 | C-terminally His-tagged FilP expression in *S. coelicolor* | This study |  |
